# The Interplay Between Lifestyle and Oral/Faecal Microbial Profiles Among Periodontal Disease Patients: A Cross‐Sectional Study

**DOI:** 10.1111/jcpe.70029

**Published:** 2025-09-07

**Authors:** Marcella Costa Ribeiro, Ana Paula Vieira Colombo, Adriana Miranda de Oliveira, Talita Gomes Baêta Lourenço, Heitor Marques Honório, Ellen Cristini de Freitas, Michel Reis Messora, Flávia Aparecida Chaves Furlaneto

**Affiliations:** ^1^ Department of Oral and Maxillofacial Surgery and Periodontology Ribeirao Preto School of Dentistry, University of Sao Paulo (USP) Ribeirao Preto Brazil; ^2^ School of Dentistry, Postgraduate Program in Periodontics Federal University of Rio de Janeiro Rio de Janeiro Brazil; ^3^ Laboratory of Oral Microbiology, Department of Medical Microbiology Institute of Microbiology Rio de Janeiro Brazil; ^4^ Department of Pediatric Dentistry, Orthodontics and Public Health Bauru School of Dentistry, University of São Paulo Bauru Brazil; ^5^ School of Physical Education and Sport of Ribeirão Preto University of São Paulo (USP) Ribeirão Preto Brazil

**Keywords:** intestinal microbiome, metagenome, microbiota, periodontal diseases

## Abstract

**Aim:**

To characterise periodontal and faecal microbiomes of individuals with periodontal health (PH) and diseases, and evaluate associations with periodontal, sociodemographic, anthropometric, nutritional and lifestyle factors.

**Materials and Methods:**

Dental biofilm and faecal samples from individuals (*n* = 24/group) with PH, gingivitis (GG) and periodontitis (PE) were sequenced (16S rRNA). Anthropometric data and questionnaires on demographics, lifestyle, diet and intestinal habits were collected. Data were statistically analysed (*p* < 0.05).

**Results:**

GG and PE groups showed higher age, BMI, waist/abdominal circumferences and trans‐fat intake and lower selenium and vitamin E intake compared to PH. Individuals with PE had higher hip circumference and lower income, education and intake of iron as well as vitamins A and B9. PE microbiomes (oral and faecal) showed distinct compositions, with the highest number of unique oral species. Faecal richness was lower in PE and GG compared to PH. Specific microbial taxa correlated with periodontal status and host factors.

**Conclusion:**

Periodontal and faecal microbiomes vary across periodontal conditions. Discriminant analysis classified 77% of individuals by periodontal status, with key markers for PE including older age, poor dietary quality and distinct microbial oral and faecal signatures. These findings highlight the role of clinical, dietary and microbial factors in periodontal disease profiling.

## 1 Introduction

Periodontitis (PE) is a prevalent, polymicrobial immunoinflammatory disease with increasing incidence among younger populations (Wu, Yang, et al. 2022; Wu, Zhang, et al. 2022). Oral bacteria can disseminate systemically (Dewhirst et al. 2010), linking PE to various systemic conditions (Genco and Sanz 2020), including inflammatory bowel disease (IBD) (She et al. 2020). The intestinal microbiome impacts both intestinal and extra‐intestinal inflammatory diseases (Vijay and Valdes 2022), several of which also are related to PE (Genco and Sanz 2020). Interest in the mouth–gut axis has increased (Tan et al. 2023), with evidence suggesting that beyond bacteremia and metastatic inflammation (Hajishengallis 2015), microbial transmission along this axis may alter both oral and intestinal ecosystems, contributing to diseases (Tan et al. 2023; Park et al. 2021).

PE may induce intestinal dysbiosis by facilitating the translocation of periodontopathogens and disrupting immune responses (Arimatsu et al. 2014; Kato et al. 2018; Komazaki et al. 2017; Liu et al. 2022). In mice, PE was found to aggravate colitis through oral pathobiont translocation (e.g., *Enterobacteriaceae*) and immune activation (Th17 responses), supporting a ‘multiple‐hit’ hypothesis for oral–intestinal inflammation (Kitamoto et al. 2020). Clinical studies have shown intestinal dysbiosis in advanced PE patients (Amado et al. 2020; Kawamoto et al. 2021), and increased oral bacteria in faecal samples of patients with IBD suggest ectopic colonisation during gut inflammation (Golob et al. 2023; Imai et al. 2021). Conversely, a dysbiotic intestinal microbiota may contribute to oral microbiome imbalance through systemic hyperinflammation, aligning with the ‘ecological plaque hypothesis’ and the concept of ‘inflammation control for infection control’ (Bartold and Van Dyke 2017; Marsh 2003; Marsh et al. 2011). IBD patients exhibit greater prevalence, severity and extent of PE (Brito et al. 2008; Habashneh et al. 2012), possibly due to microbial (Docktor et al. 2012; Said et al. 2014) and immunological mechanisms (Schmidt et al. 2018).

Host factors such as age, genetics, lifestyle, dietary patterns, body mass index (BMI), inflammatory profile and antimicrobial use influence oral and intestinal microbiomes (Aumeistere et al. 2022; Vujkovic‐Cvijin et al. 2020; Zaura et al. 2021). While previous studies have evaluated oral and intestinal microbiomes in different periodontal conditions, they often used limited sample sizes and/or saliva (Amado et al. 2020; Baima et al. 2024; Bao et al. 2022; Kawamoto et al. 2021; Lourenço et al. 2022; Miyauchi et al. 2025). While salivary samples are useful for monitoring oral health (Kageyama et al. 2017), dental biofilm samples may offer a more precise representation of the periodontal microbiota. Notably, they can differ from salivary samples, particularly in terms of the site‐specific microbial communities associated with periodontal destruction (Abusleme et al. 2021; Acharya et al. 2019; Amado et al. 2020). Although some studies have evaluated the oral and faecal microbiomes in individuals with periodontal health (PH) and diseases, to the best of our knowledge, this is the first study to comprehensively characterise these microbiomes—using dental biofilm samples for the periodontal microbiome—while simultaneously exploring their interrelationship with a wide range of clinical, anthropometric, sociodemographic, lifestyle and, notably, nutritional parameters. Additionally, this study aims to identify classification profiles capable of distinguishing between periodontal clinical conditions based on multifactorial associations, as well as to uncover potential markers—particularly host‐related parameters—associated with these conditions.

## 2 Materials and Methods

### 2.1 Study Population and Sample Size

This cross‐sectional study followed Resolution 466/12 of the Brazilian CEP/CONEP system and the Declaration of Helsinki (2013). It was approved by the Ethics Committee of the Ribeirão Preto School of Dentistry, University of São Paulo (Protocol No. 40573820.8.0000.5419), and all participants provided informed consent. Sample size was based on expected differences in gut microbiota diversity (Shannon index) between individuals with PH and PE, using a power of 80% and α = 0.05, accounting for a 15% dropout rate. A total of 72 adults (≥ 18 years, ≥ 15 teeth, good general health) were recruited between July 2021 and April 2022 and categorised into PH, gingivitis (GG) or PE groups. Periodontal assessments were performed by a calibrated examiner (Appendix S1).

### 2.2 Study Design

At baseline, participants completed a food frequency questionnaire (FFQ); sociodemographic, lifestyle and bowel habits questionnaires; and a 3‐day food diary. Periodontal and anthropometric measurements were collected. Stool collection kits were provided for at‐home sampling. After 7 days, stool samples were returned, and supragingival and subgingival biofilm samples were collected (Appendix S1).

### 2.3 Dental Biofilm and Faecal Microbiological Analyses

Dental biofilm samples were collected from eight interproximal sites per individual using sterile curettes. GG and PH samples were collected from sites with and without bleeding on probing (BOP), respectively; PE samples included both healthy and diseased sites. Faecal samples were collected at home and delivered within 24 h. DNA was extracted, and sequencing of the 16S rRNA gene (oral V1–V3, faecal V3–V4) was performed. Detailed protocols are given in Appendix S1.

### 2.4 Statistical Analysis

Analyses were performed using SigmaPlot 12.0, SPSS 21 and JAMOVI 2.4.7. Periodontal, sociodemographic, anthropometric, dietary and lifestyle data were summarised descriptively. Normality was assessed using the Shapiro–Wilk test. Group differences and associations were evaluated using ANOVA, Kruskal–Wallis, Mann–Whitney, Chi‐square, MANCOVA and Spearman correlation, as appropriate, with post hoc tests (Tukey or Dunn). Microbial taxa (≥ 10% frequency and ≥ 0.1% mean abundance) were used to compute diversity metrics. Alpha diversity was assessed via Kruskal‐Wallis and Mann–Whitney tests, and beta diversity using PERMANOVA. Microbial structure was visualised with QIIME2 Emperor. Stepwise multiple discriminant analysis (MDA) was applied to predict periodontal status using oral and gut taxa, along with host factors. Variables were log‐transformed and standardised. Statistical significance was set at 5%. Detailed methods are given in Appendix S1.

## 3 Results

### 3.1 Periodontal, Sociodemographic, Anthropometric and Lifestyle Characteristics of the Population

Of the 158 individuals screened, 72 were included (Figure S1). PE and GG individuals showed higher age and anthropometric measures and lower income (*p* < 0.05; Table 1).

**TABLE 1:** Periodontal, sociodemographic, anthropometric and lifestyle parameters of the study population.

Parameters	PH (*n* = 24)	GG (*n* = 24)	PE (*n* = 24)	*p*
PI (%), mean (SD)	14.16 (8.12)^a^	57.82 (4.70)^b^	66.10 (14.60)^b^	< 0.001*‡
BOP (%), mean (SD)	7.58 (2.28)^a^	62.21 (17.46)^b^	81.79 (17.8)^b^	< 0.001*‡
PPD (mm), mean (SD)	2.10 (0.23)^a^	2.37 (0.18)^a^	3.44 (0.5)^b^	< 0.001°Ϟ
CAL (mm), mean (SD)	1.33 (0.28)^a^	1.32 (0.48)^a^	3.27 (0.84)^b^	< 0.001*‡
*N* of teeth, mean (SD)	28.01 (2.21)^a^	26.30 (2.33)^b^	27.20 (3.15)^ab^	< 0.05*‡
Age (years), mean (SD)	26.2 (8.30)^a^	36.50 (14.30)^b^	43.20 (10.70)^b^	< 0.001*‡
Abdominal circumference (cm), mean (SD)	76.00 (6.20)^a^	83.60 (11.60)^b^	83.30 (8.10)^b^	= 0.008*‡
Waist circumference (cm), mean (SD)	73.40 (8.90)^a^	81.40 (12.00)^b^	85.30 (9.10)^b^	< 0.001°Ϟ
Hip circumference (cm), mean (SD)	96.20 (6.20)^a^	101.10 (10.40)^ab^	103.20 (9.02)^b^	= 0.022°Ϟ
BMI (kg/m^2^), mean (SD)	23.10 (2.80)^a^	25.60 (4.00)^b^	26.40 (3.00)^b^	= 0.003*‡
Gender (%)				> 0.05
Females	70.8	70.8	75.0	
Males	29.2	29.2	25.0	
Race (%)				> 0.05
White	75.0	66.7	58.3	
Black	8.3	12.5	16.7	
Multiracial	16.7	20.8	25.0	
Education, median (IQR)	2.0^a^ (2.0 to –3.0)	2.0^a^ (2.0 to –3.0)	2.0^b^ (2.0 to –2.0)	= 0.02*‡
Monthly family income, median (IQR)	3.0^a^ (2.0 to 3.0)	2.0^ab^ (1.0 to –3.0)	1.0^b^ (1.0 to –2.0)	= 0.005*‡
Physical activity, median (IQR)	2.0 (1.0 to –2.0)	2.0 (1.0 to –3.0)	1.0 (1.0 to –2.0)	> 0.05
Teeth cleaning, median (IQR)	3.0 (2.0 to –30)	3.0 (2.0 to –3.0)	3.0 (3.0 to –3.0)	> 0.05

*Note*: Significant differences among groups (*Kruskal‐Wallis and °ANOVA), and between pairs of groups (‡ Dunn test and Ϟ Tukey test, *p* < 0.05). Different letters indicate significant difference between groups. Education was categorized into elementary school (1), high school (2), and higher education (3). Family income was categorized into < 2 minimum wages (1), 2‐3 minimum wages (2), and > 3 minimum wages (3). Physical activity was categorized into sedentary (0‐2 days/week), active (3‐4 days/week), and very active (≥ 5 days/week). Tooth cleaning was categorized into once a day (1), twice a day (2), and three or more times a day (3).

Abbreviations: BMI, body mass index; BOP, bleeding on probing; CAL, clinical attachment level; GG, gingivitis; IQR, interquartile range; *N*, number; PE, periodontitis; PH, periodontal health; PI, plaque index; PPD, probing pocket depth; SD, standard deviation.

### 3.2 Dietary Data and Bowel Habits

**FIGURE 1:**
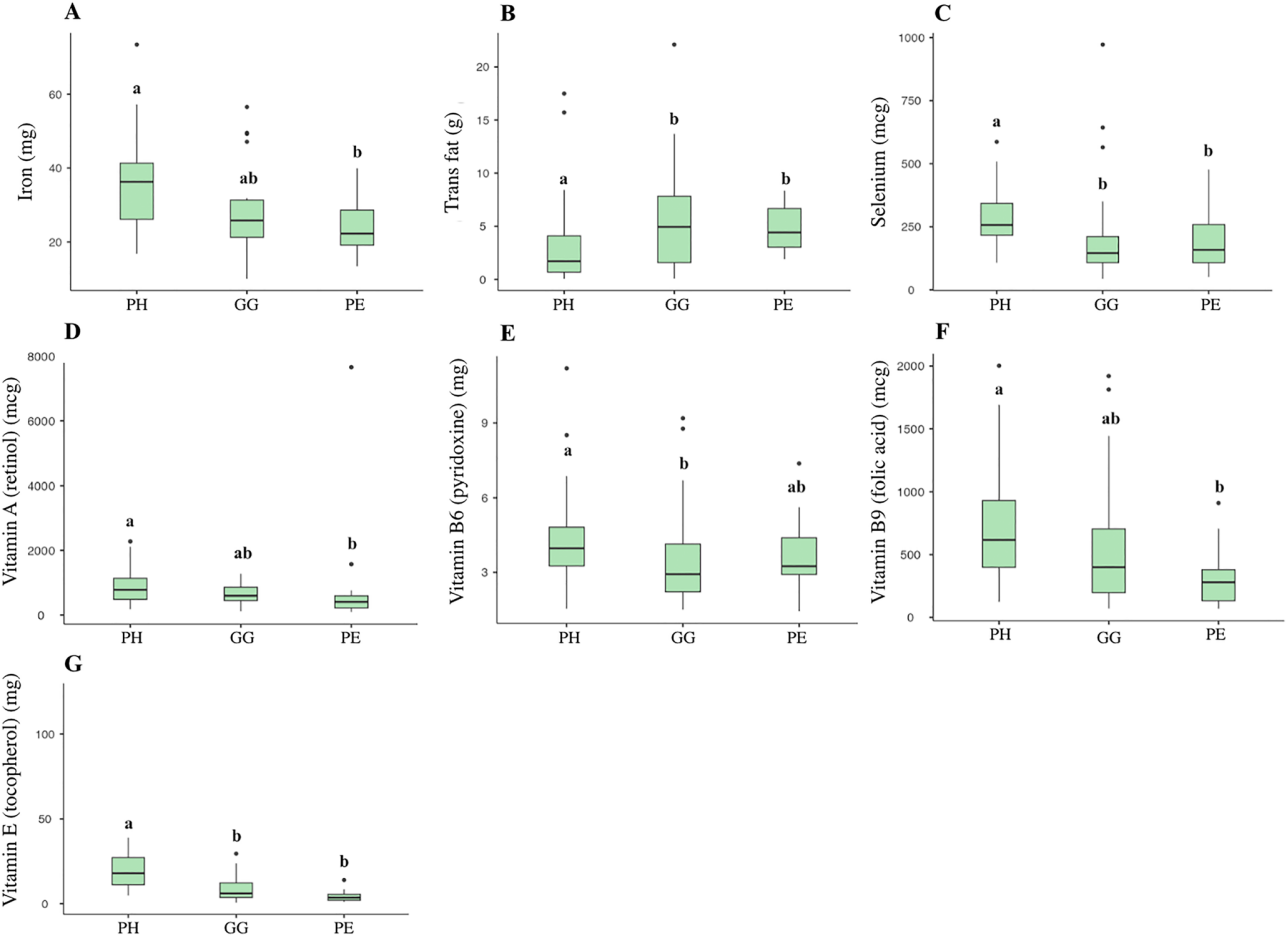
Intake of iron (2A), trans fat (2B), selenium (2C), vitamin A (2D), vitamin B6 (2E), vitamin B9 (2F) and vitamin E (2G) from the 3‐day food record in each of the clinical groups. PH, periodontal health; GG, gingivitis; PE, periodontitis. Boxes represent the median, 25th and 75th quartiles. Whiskers represent the interquartile range of 1.5; circles represent outliers in each group. Significant differences between the three groups (Kruskal–Wallis test, *p* < 0.05). Different letters indicate significant differences between pairs of groups (Dunn's test, *p* < 0.05).

FFQ data and bowel habits showed no significant differences between groups (*p* > 0.05; Table S1). However, 3‐day food records indicated that PH individuals had a higher intake of vitamin E, selenium (vs. GG and PE), vitamin B6 (vs. GG), iron, vitamin A and B9 (vs. PE). GG and PE groups reported higher trans‐fat intake than the PH group (*p* < 0.05; Figure 1).

### 3.3 Diversity of the Oral–Faecal Microbiota

**FIGURE 2:**
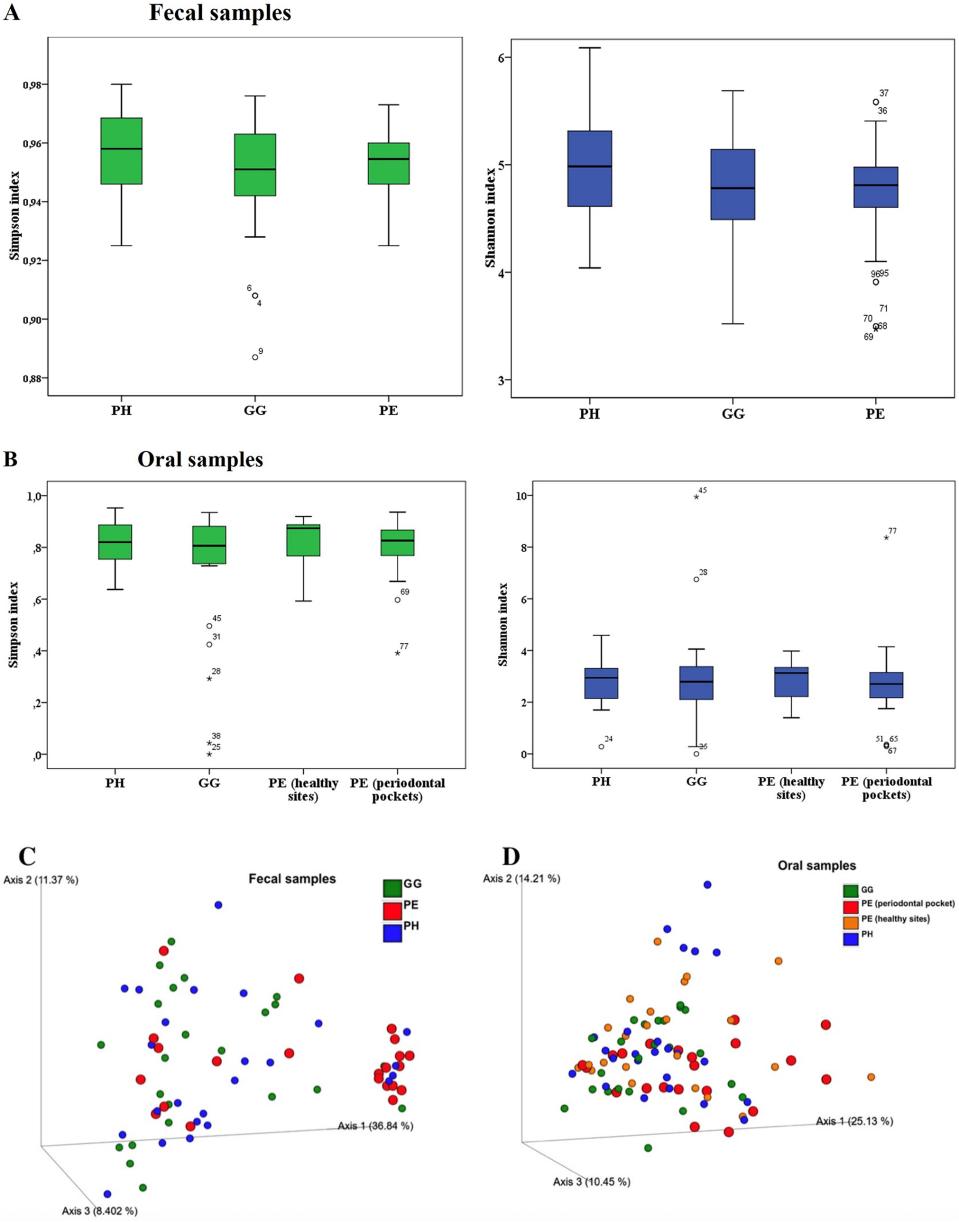
Alpha‐diversity of microbial taxa in oral biofilm and faecal samples from the periodontal health (PH), gingivitis (GG) and periodontitis (PE) groups, including periodontal pockets and healthy sites in PE patients. The boxes represent the median, 25th and 75th quartiles. Whiskers represent the interquartile range of 1.5; circles and asterisks represent outliers in each group. No differences were observed among the clinical groups for faecal (A) or oral (B) microbiota diversity as assessed by Simpson and Shannon indices, controlling for age and BMI (Kruskal–Wallis test, *p* > 0.05). The principal coordinates analysis (PCoA) plot, using the weighted UniFrac distances, demonstrates the beta diversity of faecal (C) and oral (D) microbiota among the three clinical groups at the species/phylotype level. The faecal microbiota of the PE group was distinct from those of PH and GG groups (*p* < 0.05, PERMANOVA). For the biofilm microbiota, sites with periodontal pockets were significantly distinct from sites with gingivitis (*p* = 0.007) and periodontal health (*p* = 0.001), as well as healthy sites from periodontitis patients (*p* = 0.015; PERMANOVA).

Alpha‐diversity (Simpson and Shannon indices) showed no significant differences after controlling for age and BMI (Figure 2A,B), although a progressive reduction in faecal diversity was observed from PH to PE. Beta‐diversity (weighted UniFrac) revealed that faecal microbiota from PE differed significantly from GG (*p* = 0.009) and PH (*p* = 0.03), but not between PH and GG (*p* > 0.05; Figure 2C). Oral biofilm from PE pockets was distinct from those from PH (*p* = 0.001), GG (*p* = 0.007) and healthy PE sites (*p* = 0.015), while no differences were observed between healthy PE sites, PH and GG (*p* > 0.05; PERMANOVA; Figure 2D).

### 3.4 Oral–Faecal Microbiota Composition

**FIGURE 3:**
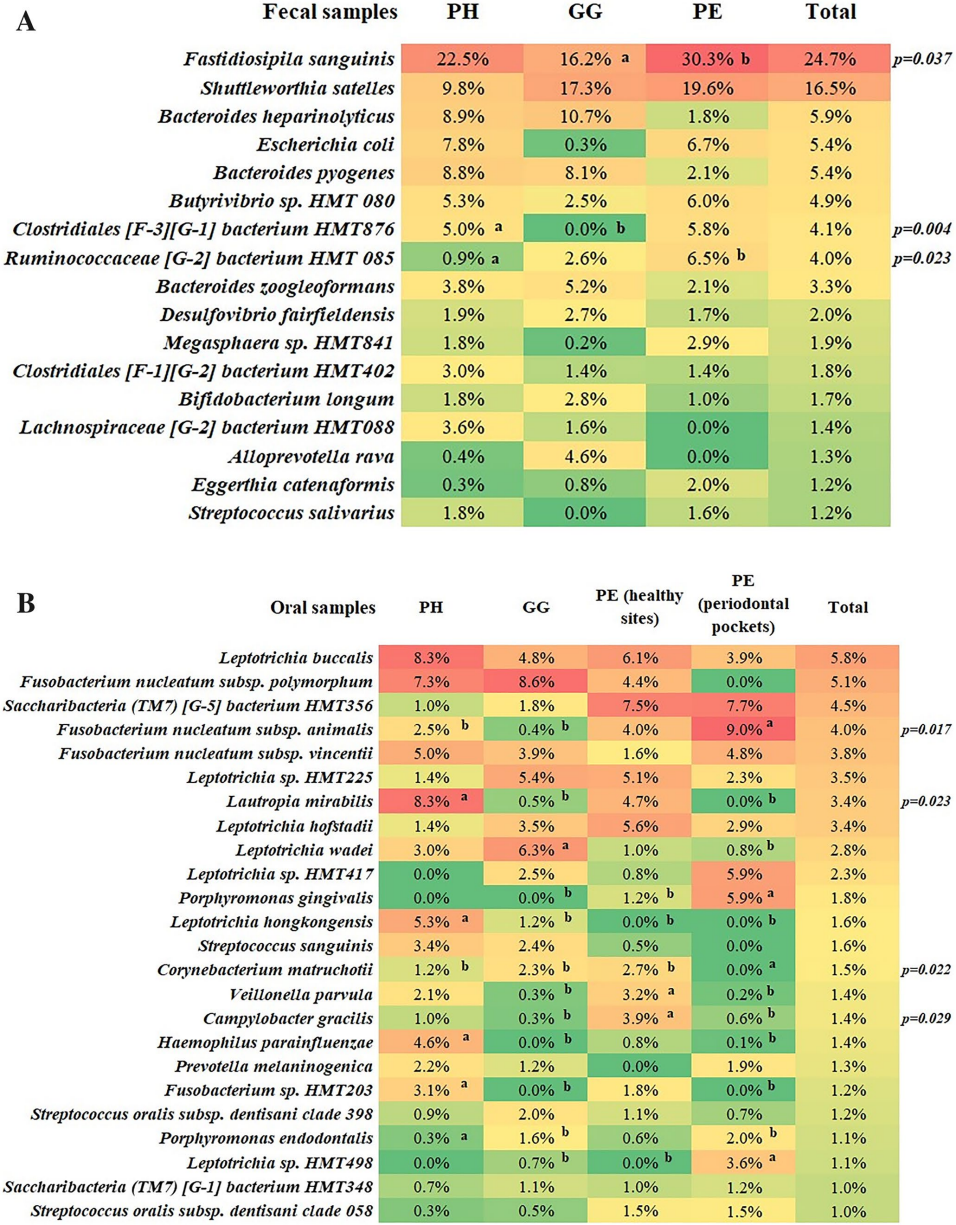
Mean relative abundance of predominant bacterial taxa (≥ 1.0% of total samples) at the species and phylotype levels in faecal (A) and dental biofilm (B) samples from individuals with PH (periodontal health), GG (gingivitis) and PE (periodontitis, including healthy sites and periodontal pockets). Taxa are ranked according to abundance. Different letters refer to significant differences between group pairs (Mann–Whitney test). Significant differences were observed for a few taxa after adjusting for multiple comparisons (Bonferroni test) and controlling for age, income, education as well as nutritional and anthropometric parameters (*p* < 0.05, MANCOVA).

**FIGURE 4:**
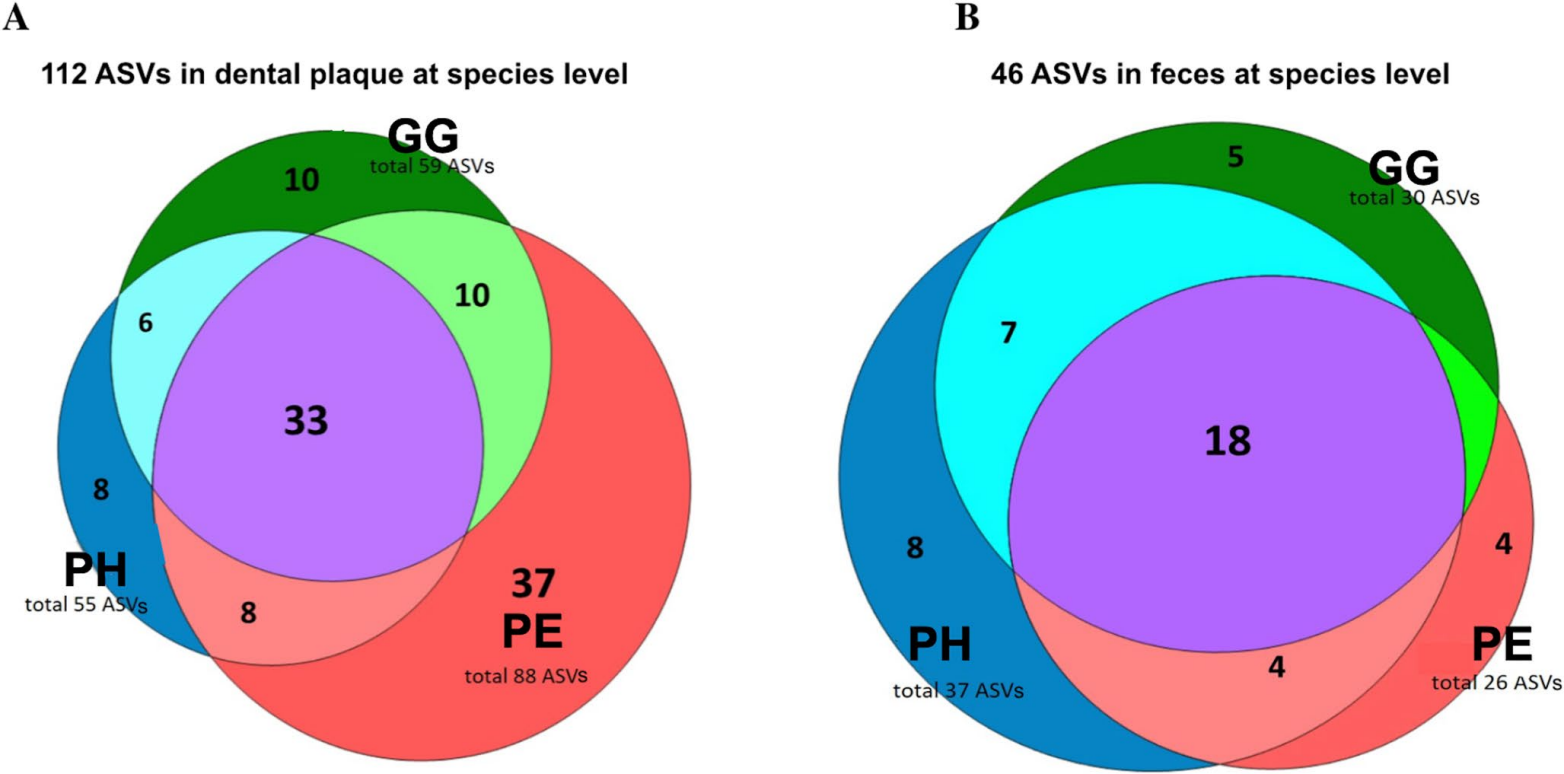
Venn diagram indicating the number of ASVs, at the species/phylotype level, shared among the clinical groups.

Detailed descriptions are available in Figure S2 and Figure 3A,B and Appendix S1. Of 112 oral and 46 faecal most abundant amplicon sequence variants (ASVs), 29.5% and 39% were shared between the groups, respectively. Considering the species detected in this cohort, PE had more unique oral species (33%) than PH or GG (Figure 4A,B). In faeces, PH individuals had nearly twice as many unique species as the periodontal disease groups, suggesting lower richness in the latter.

### 3.5 Identification of Species Preferentially Colonising the Oral Microbiota in Faecal Samples

To identify species with a preference for colonising the oral microbiota in faeces, 47 species matching the expanded Human Oral Microbiome Database (eHOMD) 16S rRNA Reference Sequence Version 15.23 database were evaluated and confirmed with BacDive. Of these, 18 were found in PH, 17 in GG and 14 in PE, with no significant difference between groups.

### 3.6 Distinct Oral and Faecal Taxa Correlate With Periodontal, Sociodemographic, Anthropometric, Nutritional and Lifestyle Parameters

Spearman correlation (> 0.500, *p* < 0.01; Table 2) revealed the associations between microbial taxa and host variables. In PH, higher faecal abundance of *Megasphaera* sp. HMT 841 was associated with reduced probing depth and attachment loss, while *Lachnospiraceae [G‐2] bacterium* HMT 088 was associated with higher education and vitamin B6 intake. In PE, *Megasphaera* sp. HMT 841 was linked to lower education. In oral samples, *Lautropia mirabilis* (PH) correlated with shallower probing depths and lower trans‐fat intake despite a positive correlation with BMI. In PE, *Porphyromonas endodontalis* negatively correlated with education.

**TABLE 2:** Spearman's correlation (rho) analysis between periodontal clinical, anthropometric, sociodemographic and nutritional parameters, and phylotypes/species detected in faecal and dental biofilm samples.

	PPD	CAL	REC	PI	Age	Income	Education	Abdominal circumference	BMI	Fat trans	Selenium	Vit A	Vit B6	Vit B9
FAECAL TAXA														
PH														
*Megasphaera* sp. *HMT841*	**−0.588**	**−0.586**												
*Butyrivibrio* sp. *HMT080*								**−0.617**						
*Escherichia coli*					**−0.574**				**−0.556**					
*Clostridiales [F‐3][G‐1] bacterium HMT876*								**0.532**						
*Bacteroides pyogenes*					**−0.544**									
*Desulfovibrio fairfieldensis*			**−0.531**											
*Lachnospiraceae [G‐2] bacterium HMT088*							**0.560**						**0.573**	
GG														
*Shuttleworthia satelles*												**−0.503**		
*Bacteroides pyogenes*				**−0.608**										
*Bacteroides heparinolyticus*			**−0.500**					**−0.533**						
*Desulfovibrio fairfieldensis*														**0.508**
PE														
*Escherichia coli*												**0.550**		
*Clostridiales_[F‐3][G‐1] bacterium HMT876*												**−0.623**		
*Megasphaera* sp. *HMT841*							**−0.555**							
ORAL TAXA														
PH														
*Prevotella* sp. *HMT472*									**−0.514**					
*Leptotrichia hongkongensis*											**0.513**			
*Lautropia mirabilis*	**−0.521**								**0.503**	**−0.608**				
*Saccharibacteria (TM7) [G‐1] bacterium HMT348*														**−0.577**
*Kingella oralis*						**0.512**								
GG														
*Leptotrichia hofstadii*														
*Streptococcus oralis* subsp. *dentisani clade 398*		**−0.503**												
*Kingella oralis*		**−0.503**												
PE														
*Streptococcus oralis* subsp. *dentisani clade 398*							**0.513**							
*Porphyromonas endodontalis*							**−0.513**							

*Note*: Only *ρ* > 0.500 at *p* < 0.01were considered. Positive correlations above 0.500 are shown in bold.

Abbreviations: BMI, body mass index; CAL, clinical attachment level; GG, gingivitis; GR, gingival recession; PE, periodontitis; PH, periodontal health; PI, plaque index; PPD, pocket probing depth; REC, recession.

### 3.7 Correlation Between Oral and Faecal Taxa

Moderate to strong correlations between oral and faecal species were observed in all groups (Table S2). Detailed descriptions are available in Appendix S1.

### 3.8 Classifying Periodontal Health and Diseases Based on Oral–Gut Species, Nutritional and Sociodemographic Characteristics

**FIGURE 5:**
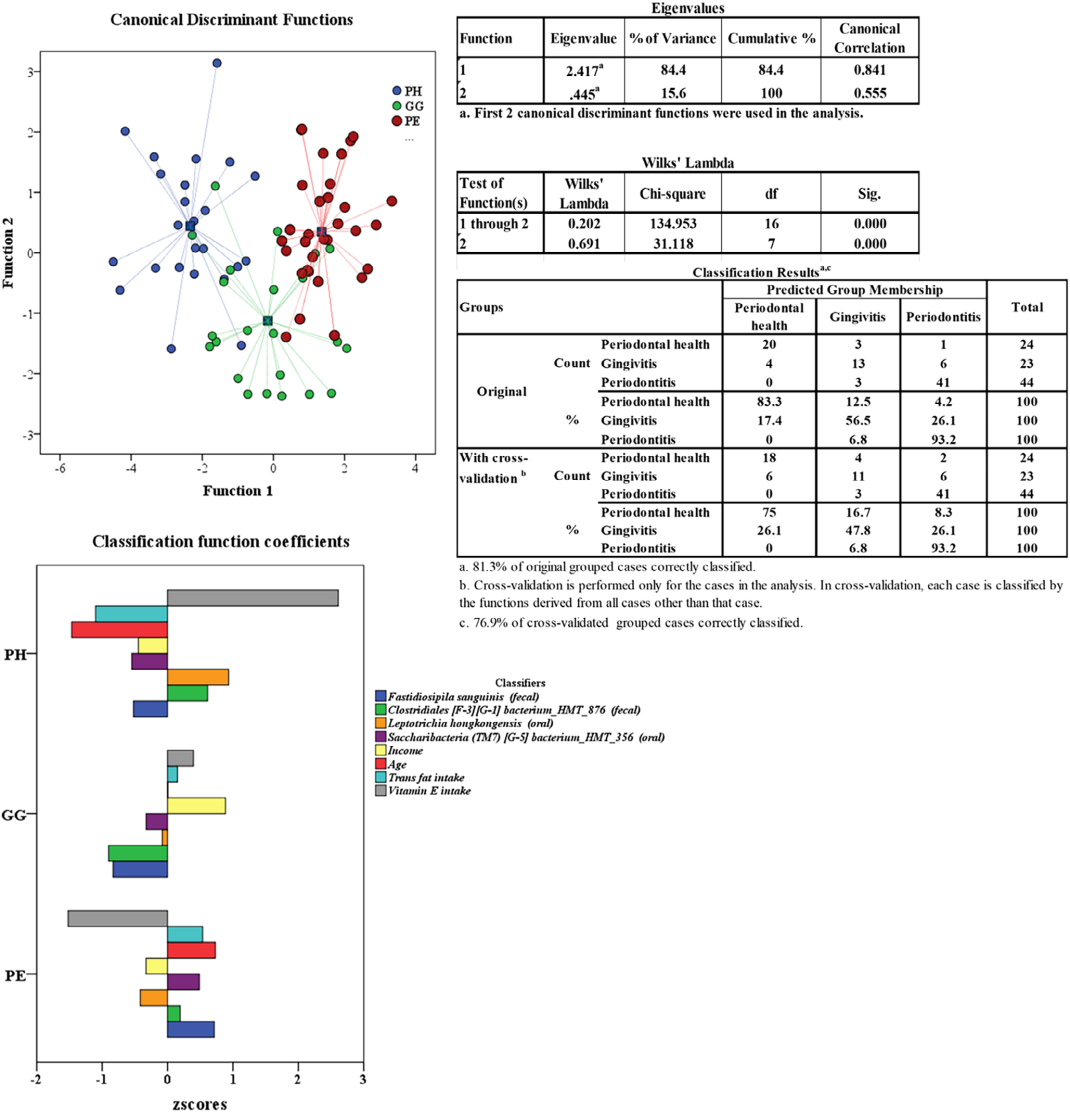
Stepwise multivariate discriminant analysis (MDA) performed to differentiate individuals with periodontal health (PH), gingivitis (GG) and periodontitis (PE) using the Mahalanobis distance metric. Out of the 70 oral–gut species/phylotypes and 13 non‐microbial predictors (age, income, anthropometric measurements, intake of iron, trans‐fat, selenium, vitamins A, B6, B9, E) entered in the model, only 8 were considered as good predictors. The first discriminant function (eigenvalue = 2.417) was significant (Wilks' λ = 0.202, χ^2^ = 134.95, *p* < 0.001), and explained 84.4% of the between‐group variance. Fisher's linear discriminant function was used to calculate the classification coefficients. The overall validated accuracy of the model was able to correctly classify 76.9% of individuals into their respective clinical groups, demonstrating the discriminative potential of integrated microbiome and host‐related data.

A stepwise MDA was conducted using 83 predictors, including oral and gut taxa, age, income, anthropometric and dietary data (Figure 5). The final model retained age, income, vitamin E and trans‐fat intake, faecal *F. sanguinis* and *Clostridiales bacterium* HMT 876 and oral *Saccharibacteria bacterium* HMT 356 and *L. hongkongensis* , explaining 84.4% of group variability (Wilks' λ = 0.202; *F* = 12.376; *p* < 0.001). PE was associated with increased age, higher trans‐fat intake, lower vitamin E intake and higher abundance of faecal *F. sanguinis* and oral *S. bacterium* HMT 356. In contrast, PH was characterised by younger age, lower trans‐fat intake, higher vitamin E intake and increased abundance of oral *L. hongkongensis* and faecal *Clostridiales bacterium* HMT 876. Low abundance of this faecal taxon was a good classifier of GG. The model correctly classified 77% of individuals overall. Performance was highest for PE (93% sensitivity, 83% specificity), followed by PH (75% sensitivity, 91% specificity). Classification accuracy for GG was lower (47.8%).

## 4 Discussion

This study revealed distinct periodontal and faecal microbiome profiles in PH, GG and PE, highlighting associations with clinical, anthropometric, sociodemographic, lifestyle and nutritional variables. To our knowledge, this is the first study to simultaneously analyse oral and faecal microbiota using biofilm and stool samples across these clinical groups, exploring their interrelationships and associations with a broad range of host factors.

Advancing age and low socioeconomic status, especially income and education, are risk indicators for PE (Darby 2022; Oppermann et al. 2015; Bastos et al. 2011). Consistently, individuals with PE in this study showed lower income and education levels than PH (*p* < 0.05). Obesity affects periodontal tissues (Jepsen et al. 2018), although its biological mechanisms remain unclear (Martinez‐Herrera et al. 2017). Previous studies have reported higher BMI in PE than in GG or PH (Lourenςo et al. 2018; Lourenço et al. 2022), which aligns with our findings: both GG and PE groups were overweight (BMI 25–29.9 kg/m^2^), with significantly higher BMI than PH. Fat distribution also contributes to systemic risk (Klein et al. 2007). In this study, GG and PE individuals had higher abdominal and waist circumferences than PH, and PE also presented greater hip circumference. These results corroborate findings from NHANES (2011–2014) and a systematic review showing waist circumference as a risk factor for PE (Liu et al. 2024; Keller et al. 2015).

Human diet influences oral microbiota, as the mouth is the first contact point for food (Liu et al. 2020; Wade 2021). Micronutrient deficiencies may increase oxidative stress and inflammation and impair collagen and bone metabolism, contributing to periodontal breakdown (Dommisch et al. 2018). Antioxidants such as vitamins E and C, β‐carotene, selenium, iron and zinc help attenuate these effects (Puertollano et al. 2011; Iwasaki et al. 2012; Muniz et al. 2015). Certain dietary patterns and supplementation with vitamins C, D, E, B‐complex, calcium and magnesium benefit periodontal outcomes (Bartha et al. 2022; Neiva et al. 2005; Woelber et al. 2019), while lower levels of vitamin A precursors have been observed in PE patients (Hans et al. 2023). Higher plasma trans‐fat levels are linked to PE in overweight/obese populations (Wu et al. 2023). In our study, individuals in the PH group consumed more vitamin E, selenium, iron, vitamin A and B9, and less trans‐fat compared to those in the GG and PE groups, reinforcing the relevance of dietary quality in PH.

No significant differences in oral alpha diversity were observed between the groups, consistent with earlier reports (Galimanas et al. 2014; Kawamoto et al. 2021; Kirst et al. 2015). However, other studies have reported either lower (Ai et al. 2017; Pérez‐Chaparro et al. 2018) or higher diversity in PE compared to healthy states (Abusleme et al. 2013; Li et al. 2014; Lourenço et al. 2022; Bao et al. 2022; Baima et al. 2024). Such discrepancies may reflect methodological differences or disease severity (Kawamoto et al. 2021).

In faeces, microbial diversity decreased from PH to PE, but without significance, matching previous studies (Kawamoto et al. 2021; Lourenςo et al. 2018; Lourenço et al. 2022; Baima et al. 2024; Bao et al. 2022). While higher gut diversity is associated with health, reduced diversity is linked to diseases (Aggarwal et al. 2022). In the dental biofilm, the PE group showed greater richness—37 unique species versus 8–10 in PH/GG group out of 112—which is a controversial finding in the literature (Abusleme et al. 2013; Li et al. 2014; Lourenço et al. 2022; Pérez‐Chaparro et al. 2018). This may reflect the increased plaque biomass in PE, favouring persistence and expansion of low‐abundance microorganisms (Abusleme et al. 2021; Amado et al. 2020; Chen et al. 2018; Kawamoto et al. 2021; Relvas et al. 2021). Conversely, faecal samples from PE and GG showed approximately half the number of unique species found in PH, indicating lower richness in periodontal diseases—similar to findings by Lourenço et al. (2022). To the best of our knowledge, this is the first study to present beta‐diversity data in GG. Periodontal pockets microbiomes clustered separately from PH, as has been previously demonstrated (Kawamoto et al. 2021), besides being dissimilar from healthy sites of PE and from GG. PE faecal microbiota also differed significantly from those of PH and GG, unlike previous studies (Kawamoto et al. 2021; Lourenço et al. 2022; Lourenςo et al. 2018).

This study identified a higher abundance of *Lautropia mirabilis* , *Corynebacterium matruchotii* , *Leptotrichia hongkongensis* , *Haemophilus parainfluenzae* , *Fusobacterium* sp. HMT203 and *Veillonella parvula* in healthy patients/sites compared to PE (periodontal pockets) and/or GG. Consistently, *H. parainfluenzae* and *L. mirabilis* have been repeatedly associated with healthy conditions (Abusleme et al. 2013, 2021; Ai et al. 2017; Bik et al. 2010; Colombo et al. 2009; Hong et al. 2015; Kistler et al. 2013). Other microorganisms frequently reported in the healthy oral environment include *Actinomyces*, *Capnocytophaga*, *Corynebacterium matruchotii* , *Rothia dentocariosa* , *Streptococcus* and *Veillonella parvula* (Curtis et al. 2020; Welch et al. 2016). These patterns may help distinguish health‐ from disease‐associated communities. Faecal *Fastidiosipila sanguinis* was enriched in PE compared to GG, consistent with its association with bacteremia and systemic inflammation (Falsen et al. 2005; Kjær Hansen et al. 2020). Although limited literature exists on *Ruminococcaceae [G‐2] bacterium* HMT 085 and *Clostridiales [F‐3][G‐1] bacterium* HMT 876, our data indicate that HMT 085 is more abundant in PE patients, whereas HMT 876 is more prevalent in PH. A recent study demonstrated persistent gut dysbiosis and serum metabolomic alterations even after periodontal therapy (Miyauchi et al. 2025), supporting long‐term systemic effects of periodontal inflammation.

There are limited and variable findings on the oral–gut microbiome connection (Baima et al. 2024; Bao et al. 2022; Kawamoto et al. 2021; Lourenço et al. 2022). This study found a restricted microbial overlap between oral and gut microbiomes in systemically healthy individuals. Methodological differences, particularly in the 16S rRNA regions analysed, may explain the discrepancies across studies. In this study, V1–V3 was used for oral samples—commonly adopted in oral microbiome research due to its phylogenetic resolution and ability to assess richness and diversity (Abusleme et al. 2013; Zheng et al. 2015)—while V3–V4 was chosen for faecal samples, in line with gut microbiome studies (Du et al. 2022; Liu et al. 2019). Although this limits direct taxonomic comparison, region‐specific choices aimed to optimise identification per habitat, acknowledging site‐specific microbiome characteristics (Zhou et al. 2024). While bacterial translocation from the oral cavity to the gut has been proposed in PE (Hajishengallis 2015; Tan et al. 2023), it is possible that oral dysbiosis indirectly influences gut microbiota via transient species or metabolites that disrupt the intestinal environment or trigger systemic inflammation, which could subsequently impact the gut microbiota (Gatej et al. 2020). Preclinical models have shown that PE can promote gut dysbiosis (Arimatsu et al. 2014; Nakajima et al. 2015), impair intestinal microstructure (Messora et al. 2013) and compromise barrier integrity (Feng et al. 2020).

Significant correlations between oral and faecal taxa across clinical groups may suggest a potential interplay between the oral and gut microbiomes. Oral *Porphyromonas endodontalis* exhibited group‐specific associations with faecal species, while oral *Kingella oralis* and faecal *Escherichia coli* correlated in both PH and GG, reflecting shared ecological or systemic factors. The consistent link between oral *Leptotrichia* sp. and faecal *Slackia exigua* in PH and PE groups, but not in GG, may indicate condition‐specific translocation or immune modulation. These findings reinforce the oral–gut axis hypothesis, suggesting bidirectional microbial influences between sites and highlighting the need for further studies on their functional implications (Tortora et al. 2023).

This study also explored associations between microbial compositions and periodontal, anthropometric, socioeconomic and lifestyle parameters. Pathogenic species generally correlated with adverse indicators, while health‐associated taxa related to favourable parameters. For example, *Lautropia mirabilis* , predominantly found in healthy sites (Abusleme et al. 2013, 2021; Hong et al. 2015; Kistler et al. 2013), was associated with shallower probing depths and lower trans‐fat intake, despite a positive correlation with BMI. Conversely, oral *Porphyromonas endodontalis* , associated with endodontic (Cao et al. 2012; Gomes et al. 2005; Machado de Oliveira et al. 2000; Tomazinho and Avila‐Campos 2007) and periodontal infections (Bedran et al. 2012; Colombo and Tanner 2019), negatively correlated with the educational level. In the PH group, faecal *Megasphaera* sp. *HMT 841* abundance was associated with better periodontal parameters. While specific literature on this species is scarce, *Megasphaera* is known to ferment carbohydrates in the gut, producing short‐chain fatty acids (SCFAs) (Shetty et al. 2013) that contribute to intestinal homeostasis (Parada Venegas et al. 2019). Faecal *Lachnospiraceae [G‐2] bacterium HMT 088* was associated with higher education and vitamin B6 intake. Despite limited characterisation, such species may contribute to host health, warranting further investigation.

An exploratory analysis integrating oral–gut taxa with sociodemographic and lifestyle parameters achieved an overall validated 77% accuracy in distinguishing healthy from diseased individuals. PE is associated with advanced age, high trans‐fat intake, low vitamin E consumption, increased faecal *F. sanguinis* , elevated subgingival *Saccharibacteria bacterium* HMT 356 and reduced oral *L. hongkongensis* . These findings suggest that diet significantly modulates periodontal inflammation—likely via the pro‐inflammatory effects of trans‐fats and the antioxidant properties of vitamin E. Notably, *F. sanguinis* has been linked to bacteremia (Falsen et al. 2005; Kjær Hansen et al. 2020). *Saccharibacteria* (formerly TM7) can represent up to 21% of the oral microbiome in mucosal infections (Rylev et al. 2010) and are associated with GG and PE (Brinig et al. 2003; Camelo‐Castillo et al. 2015; Kistler et al. 2013; Liu et al. 2012; Nowicki et al. 2018; Paster et al. 2002; Rylev et al. 2010; Sousa et al. 2016). Notably, HMT 356 has been detected within the crevicular epithelium of PE patients (Paster et al. 2002). Additionally, a low abundance of faecal *Clostridiales bacterium* HMT 876 proved to be an effective classifier of GG, although its function remains poorly characterised. Supporting gut–oral links, a recent Mendelian randomisation study (Hang et al. 2024) identified three gut taxa with potential causal roles in GG risk.

There is no universal sample that fully represents the oral microbiome (Zaura et al. 2021). To better characterise the periodontal microbiota, supragingival and subgingival biofilm samples were used (Abusleme et al. 2021; Acharya et al. 2019; Amado et al. 2020; Lloyd‐Price et al. 2017; Relvas et al. 2021), although pooling these samples may mask site‐specific differences. Saliva is also a viable alternative (Lourenço et al. 2022). Faecal samples, while differing from mucosal microbiota, are widely used to study the gut microbiome due to their ease of collection and sufficient biomass (Tang et al. 2020; Piancone et al. 2022), despite biopsies being the gold standard (Tang et al. 2020). Both oral and intestinal microbiomes are influenced by genetic, inflammatory and environmental factors (Zaura et al. 2021; Vujkovic‐Cvijin et al. 2020), as well as by host–microbe and microbe–microbe interactions, including metabolome dynamics (Shtossel et al. 2024), which were not addressed in this study. Additionally, dietary records based on self‐report have limitations, and biochemical markers could improve micronutrient assessment.

This study suggests that periodontal diseases may be associated with alterations in gut composition, metabolic functions and intestinal barrier integrity. However, its cross‐sectional design and the complex and dynamic host–microbiome interactions (Zaura et al. 2021) are key limitations that hinder causal inferences. The exclusion of smokers, individuals with systemic diseases and those on regular medication may also reduce the generalisability of the findings to the broader periodontal population. Nonetheless, these results have implications for improving dental guidance, encouraging healthier lifestyles and informing community‐based strategies for preventing both periodontal and other chronic non‐communicable diseases.

## 5 Conclusion

Within the limits of this study, it can be concluded that the periodontal and faecal microbiomes of individuals with PH, GG and PE differ from each other. Discriminant analysis correctly classified 77% of individuals by periodontal status, with key markers for PE including older age, poor dietary quality and distinct microbial signatures in oral and faecal samples. These findings highlight the potential of integrating clinical, dietary and microbiome data for improved risk assessment and stratification in periodontal diseases.

## Author Contributions

M.C.R.: conceptualisation, clinical investigation, data collection, data curation, formal analysis, writing – original draft and writing – review and editing. A.P.V.C.: microbiological and statistical analyses, writing – review and editing. A.M.O., T.G.B.L.: microbiological and statistical analyses. H.M.H.: methodology, statistical analysis, writing – review and editing. E.C.F.: nutritional data analysis, writing – review and editing. M.R.M.: supervision, writing – review and editing. F.A.C.F.: conceptualisation, supervision, project administration, writing – review and editing.

## Conflicts of Interest

The authors declare no conflicts of interest.

## Supporting information


**Appendix S1:** Supporting Information.

**Figure S1:** Flowchart of the study design.

**Figure S2:** Relative abundance of the most prevalent phyla (≥ 1.0% of the mean relative abundance across all samples) in oral (A) and faecal (B) samples within clinical groups. The bars indicate significant differences between groups for faecal and oral samples (Kruskal–Wallis and Mann–Whitney tests, *p* < 0.01).

**Table S1:** Dietary habits data from the Food Frequency Questionnaire (FFQ) and evacuatory habits of individuals from groups PH, GG and PE.

**Table S2:** Spearman's correlation (rho) analysis between oral and faecal bacterial species in each clinical group. (a) Significant correlations (*ρ* > 0.500; *p* < 0.01)—periodontal health (PH). (b) Significant correlations (𝜌 > 0.500; *p* < 0.01)—gingivitis (GG). (c) Significant correlations (*ρ* > 0.500; *p* < 0.01)—periodontitis (PE).

## Data Availability

The data that support the findings of this study are available from the corresponding author upon reasonable request.
