# Supplementary material for: The Interplay Between Lifestyle and Oral/Faecal Microbial Profiles Among Periodontal Disease Patients: A Cross‐Sectional Study
Source: J Clin Periodontol. 2025 Sep 7;53(1):82–97. doi: 10.1111/jcpe.70029 (PMC12695455; doi:10.1111/jcpe.70029)
Supplement: Supplementary file 1 — Appendix S1: Supporting Information. [file JCPE-53-82-s001.docx]

**Appendix File 1 of the manuscript "The Interplay Between Lifestyle and Oral/Fecal Microbial Profiles Among Periodontal Disease Patients: A Cross-Sectional Study"**

Marcella Costa Ribeiro ^1^, Ana Paula Vieira Colombo ^2,3^, Adriana Miranda de Oliveira ^2,3^, Talita Gomes Baêta Lourenço ^2^, Heitor Marques Honório ^4^, Ellen Cristini de Freitas ^5^, Michel Reis Messora ^6^, Flávia Aparecida Chaves Furlaneto ^6^

^1​​^Department of Oral and Maxillofacial Surgery and Periodontology, Ribeirao Preto School of Dentistry, University of Sao Paulo – USP, Ribeirao Preto / SP, Brazil.

^2^School of Dentistry, Postgraduate Program in Periodontics, Federal University of Rio de Janeiro, Rio de Janeiro, Brazil.

^3^Laboratory of Oral Microbiology, Department of Medical Microbiology, Institute of Microbiology, Rio de Janeiro, Brazil.

^4^Department of Pediatric Dentistry, Orthodontics and Public Health, Bauru School of Dentistry, University of São Paulo, Bauru, SP, Brazil.

^5^School of Physical Education and Sport of Ribeirão Preto, University of São Paulo-USP, Av. Bandeirantes 3900, Ribeirão Preto, SP, Brazil.

**Correspondence:**

Flávia A. C. Furlaneto

Av. Café s/n Ribeirão Preto-SP 14020-150

Telephone number: +55 16 3315 4135

Fax number: +55 16 3315 4788

Email address: [flafurlaneto@usp.br](mailto:flafurlaneto@usp.br)

**MATERIALS AND METHODS**

***Ethical Approval and Reporting Guidelines***

This study was conducted in accordance with Brazilian regulations for research involving human subjects, specifically the guidelines of the CEP/CONEP system (National Research Ethics Commission), as outlined in Resolution No. 466/12. The research also complied with the ethical principles of the Declaration of Helsinki (2013). The study protocol was approved by the Research Ethics Committee of the Ribeirão Preto School of Dentistry – University of São Paulo (Protocol No. 40573820.8.0000.5419). All participants provided written informed consent prior to enrollment. The study adhered to the STROBE (Strengthening the Reporting of Observational Studies in Epidemiology) statement for cross-sectional studies.

***Sample Size Calculation***

The sample size was calculated based on differences in gut microbiota diversity (Shannon index) between individuals with periodontal health (PH) and periodontitis (PE), using previously published data (Lourenço et al., 2018). A mean difference of 0.8 and standard deviation of 1.0 were assumed. Considering 80% power, α = 0.05, and an estimated 15% dropout rate, a total sample size of 72 individuals (24 per group) was determined.

***Study Population***

Inclusion criteria were being systemically healthy, being ≥18 years old, having at least 15 natural teeth, excluding third molars and teeth indicated for extraction, and being willing to adhere to the study protocol. In the PH group, individuals were required to have no loss of attachment or bone loss due to periodontitis, probing depth (PD) of up to 3 mm at all sites, and bleeding on probing (BoP) in up to 10% of sites in the entire oral cavity. For the GG group, participants should have generalized gingivitis, with more than 30% of sites in the entire mouth exhibiting BoP, all sites with PD of up to 3 mm, and no loss of attachment or bone loss due to periodontitis. In the PE group, participants should have generalized stage III periodontitis, grade B or C, and at least 30% of teeth with at least 1 site with PD and clinical attachment level (CAL) ≥ 5 mm and BoP (Chapple et al., 2018; Papapanou et al., 2018). Sites classified as “healthy” in individuals with PE were defined as those with probing depth ≤ 3 mm, absence of BoP, and with or without clinical attachment loss. Conversely, sites classified as “diseased” in individuals with PE were defined as those presenting PD ≥ 5 mm, presence of BoP, and clinical attachment loss.

Exclusion criteria included: use of antibiotics, probiotics, and/or prebiotics in the last six months; undergoing periodontal treatment in the last six months; continuous use of mouthwashes containing antimicrobial agents; long-term use of anti-inflammatory or immunosuppressive medication; any systemic involvement that may interfere with the onset and/or progression of periodontal diseases (e.g., diabetes, obesity, immune disorders); chronic gastrointestinal diseases; extensive prosthetic involvement; need for antibiotic prophylaxis for routine dental procedures; smoking; pregnancy and lactation.

***Calibration and blinding of examiners***

A single examiner (M.C.R.), trained and calibrated, a specialist in Periodontology, was responsible for performing all periodontal clinical measurements and dental biofilm sample collections. To achieve acceptable intra-examiner reproducibility, calibration involved ten participants, not involved in the study, who presented at least 5 teeth with PD and CAL ≥ 5 mm on interproximal surfaces. These participants were evaluated by the examiner on two separate occasions, with a 48-hour interval between them. In these evaluations, PD and CAL were measured. In the intra-examiner calibration, the Intraclass Correlation Coefficient (ICC) value for PD was 0.993 and for CAL was 0.967. The researchers who performed the next-generation sequencing analyses were blinded to the experimental group to which each individual belonged.

***Assessment of periodontal clinical parameters***

The periodontal clinical parameters were measured using a North Carolina millimeter periodontal probe (Hu-Friedy, Chicago, IL, USA) and included: (i) presence/absence of visible supragingival biofilm on four surfaces of each tooth, assessed dichotomously, with the plaque index (PI) determined by the percentage of tooth surfaces with biofilm deposits detected by gently sliding the probe over the cervical area; (ii) probing depth (PD) in mm, measured from the gingival margin to the bottom of the periodontal pocket or gingival sulcus; (iii) bleeding on probing (BoP), assessed dichotomously, with bleeding considered positive if it occurred within 20 seconds of probe insertion for PD measurement; (iv) clinical attachment level (CAL) in mm, measured from the cementoenamel junction (CEJ) to the bottom of the periodontal pocket or gingival sulcus; (v) distance from the CEJ to the gingival margin. Except for the presence/absence of supragingival biofilm, all measurements were taken at six sites per tooth (mesiobuccal, buccal, distobuccal, mesiolingual, lingual, and distolingual).

***Dietary Data Collection***

The dietary intake of participants was assessed using 3-day food records, including two weekdays and one weekend day. Participants detailed all foods and beverages consumed, noting preparation methods, ingredients, brands, portion sizes, and meal times. Dietbox® software (Dietbox Informática Ltda., Porto Alegre, Brazil) was used to calculate caloric intake, macronutrients, micronutrients, and dietary fibers (Nascimento et al., 2021). Additionally, a food frequency questionnaire (FFQ), adapted from the SISVAN dietary intake markers questionnaire (Brazil, 2008; Zanchim et al., 2018), was used. Participants reported their consumption patterns over the past seven days, covering twelve food groups: raw salad; cooked vegetables; fresh fruit or fruit salad; beans; milk or yogurt; potato chips, packaged, fried snacks; salty crackers or packaged snacks; hamburgers and processed meats; sweets in general; soda; red and/or white meat; and alcoholic beverage. Participants were instructed to maintain their usual eating habits throughout the study.

***Assessment of Anthropometric Measurements***

Height (H) measurements were taken using an inelastic measuring tape (Sekich, São Paulo, SP, Brazil), fixed to a smooth wall, and body weight (W) was measured using an electronic platform scale (Planeta Ferramentas e Utilidades Domésticas Comércio Importação e Exportação LTDA., Cândido Mota, São Paulo, Brazil). From these values, the Body Mass Index (BMI) was calculated using the following equation (WHO, 1995): BMI = W / H^2 (kg/m^2). Circumference measurements of the waist, hip, and abdomen were taken in centimeters (cm) using a sturdy, non-stretchable measuring tape, 1 cm wide, comfortably wrapped around the individual but not tight enough to constrict. The tape was kept level and parallel to the ground at all measurement points. Participants stood erect with arms relaxed at their sides, feet evenly spaced apart, and weight evenly distributed. Waist circumference was measured at the end of several consecutive natural breaths, at the midpoint between the top of the iliac crest and the bottom edge of the last palpable rib in the mid-axillary line, at a level parallel to the ground (WHO, 2011). Hip circumference was measured at the widest part of the buttocks (WHO, 2011), and abdominal circumference was measured at the maximum extension of the abdomen region (Callaway et al., 1988), both also at a level parallel to the ground.

***Dental biofilm and fecal microbiological analyses***

Supragingival and subgingival biofilm samples were collected from non-contiguous interproximal sites using sterile periodontal curettes (Hu-Friedy) and were pooled together in Tris-EDTA buffer. In the GG and PH groups, sampling was collected at 8 sites with and without bleeding on probing (BOP), respectively. In the PE group, samples were collected from 8 sites with probing depth (PD) of up to 3 mm and without BOP (healthy sites), as well as from 8 sites with the highest PD and presence of BOP (diseased sites). For fecal samples, subjects were given a sterile stool collection kit for at-home sampling. This kit comprised a collector, spatula, sanitary seat cover (ColOFF®, São Paulo, Brazil), along with gloves to prevent contamination during collection. Samples were placed in the refrigerator post-collection and delivered to the clinical research center within 24 hours. Samples were handled in a sterilized environment and stored at −80 °C.

The genomic DNA from the samples was extracted using the QIAamp DNA Micro Kit (Qiagen, Hilden, Germany) for dental biofilm and QIAamp DNA Stool Mini Kit (Qiagen, Hilden, Germany) for fecal samples, following the manufacturer's instructions. The quantification of the extracted DNA was determined using a BioTek Epoch 2 spectrophotometer (Epoch™, Biotek® Instruments, Inc., USA) and the absorbance was measured at 260 nm and 280 nm to assess sample purity. Total DNA concentration was measured by fluorescence using a Qubit® 3.0 Fluorometer and Qubit dsDNA BR Assay kit (Thermo Fisher Scientific Inc., Waltham, Massachusetts, USA), following the manufacturer's protocol. The genomic DNA was stored at -20°C.

Next-generation sequencing was performed targeting the V1-V3 hypervariable region of the 16S rRNA gene from dental biofilm samples and the V3-V4 hypervariable region of the 16S rRNA gene from fecal samples using a high-throughput system (MiSeq Illumina, San Diego, CA, USA). Next-generation sequencing analyses were conducted by blinded examiners.

***Illumina 16S rRNA Gene Sequencing***

The aliquots containing the extracted bacterial DNA from the samples were transported in styrofoam with dry ice to the BPI Genotyping Laboratory (Botucatu, SP, Brazil), which was responsible for performing the sequencing of the 16S rRNA gene. Library preparation was conducted following the instructions of the Illumina MiSeq system (Illumina, San Diego, CA, USA). The V1-V3 hypervariable region of the 16S rRNA gene from dental biofilm samples and the V3-V4 hypervariable region of the 16S rRNA gene from fecal DNA samples were amplified. The amplification reactions of the V1-V3 and V3-V4 regions of the 16S rRNA gene were performed through real-time PCR (RT-PCR), resulting in a final volume of 20 μL, containing 10 μL of GoTaq® Green PCR Master Mix (Promega Corporation, Madison, Wisconsin, USA), 1 μL of the forward primer at 10 μM, 1 μL of the reverse primer at 10 μM, 2 μL of genomic DNA, and sufficient sterile ultrapure water for 20 μL. For V1-V3 amplification, the primers 28F and 519R were used, respectively:

- 16S-V1-V3 *Forward* 5'-TCGTCGGCAGCGTCAGATGTGTATAAGAGACAGGAGTTTGATCNTGGCTCAG-3'
- 16S-V1-V3 *Reverse* 5'-GTCTCGTGGGCTCGGAGATGTGTATAAGAGACAGGTNTTACNGCGGCKGCTG-3'

For V3-V4 amplification, primers 319F e 806R, were used, respectively:

- 16S-V3-V4 *Forward* 5'-TCGTCGGCAGCGTCAGATGTGTATAAGAGACAGCCTACGGGNGGCWGCAG-3'
- 16S-V3-V4 *Reverse* 5'-GTCTCGTGGGCTCGGAGATGTGTATAAGAGACAGGACTACHVGGGTATCTAATCC-3'

The amplification program consisted of an initial denaturation at 95°C for 5 minutes, followed by 27 cycles of denaturation at 95°C for 30 seconds for dental biofilm samples and 40 seconds for fecal samples, annealing at 55°C for 30 seconds, extension at 72°C for 40 seconds, and a final extension at 72°C for 5 minutes. The amplification reactions were conducted in a Veriti Thermal Cycler (Applied Biosystems, Waltham, Massachusetts, USA). After the amplification reaction of each sample, amplification was confirmed by agarose gel electrophoresis at 2% stained with UniSafe Dye 0.03% (v/v) (Uniscience do Brasil Indústria e Comércio de Equipamentos para Laboratório Ltda., Osasco, São Paulo, Brazil). An indexing step was performed for organization and identification of reads, where indexers were inserted into common adapters (8 bp barcodes specific to each sample). This reaction was carried out by PCR following the protocol of the Nextera XT Index kit (Illumina Inc., San Diego, California, USA). The generated libraries underwent purification steps using magnetic beads Agencourt AMPure XP (Beckman Coulter, Brea, California, USA), to remove very small fragments from the total population of molecules and primer remnants. Subsequently, quantification was performed by real-time PCR methodology using the KAPA Library Quantification Kit (Kapa Biosystems Inc., Boston, Massachusetts, USA) on the QuantStudio 3 equipment (Applied Biosystems, Waltham, Massachusetts, USA), all according to the manufacturer's protocols. Additionally, an equimolar pool of DNA was generated by normalizing all samples to 4 nM for sequencing, which was conducted using the Illumina MiSeq next-generation sequencing system and MiSeq Reagent Kit V3 600 cycles – paired-end 2 x 300 bp reading (Illumina Inc., San Diego, California, USA).

***Bioinformatics Analysis of Sequencing Data***

Demultiplexing, barcode and linker-primer sequence trimming, and initial raw read quality filtering were performed (Q score of 30, removal of reads contaminated by adapters, with ambiguous or N base calls, and those with low complexity, indicated by 10 consecutive identical bases). Read pairs were merged into unique sequences (contigs) using the Quantitative Insights into Microbial Ecology (QIIME2-2022.2) package with default settings. Samples with < 500 read counts were excluded from the QIIME2 analysis. Short reads (< 200 nt) were also removed.

Sequence quality control and amplicon sequence variant (ASV) table construction were performed with the DADA2 plugin. Phylogenetic diversity analyses were conducted using the q2-phylogeny and q2-diversity packages. For taxonomic assignment, feature classifiers were trained by q2-feature-classifier within QIIME2 (Bolyen et al., 2019). Taxonomies were assigned to unique ASVs and aligned to the Human Oral Microbiome Database of Expanded Ribosomal RNA (eHOMD 16S rRNA RefSeq Version 15.1) with 99% sequence similarity. Alpha diversity analysis was determined by the Shannon index, generated from the rarefied data distance matrix. Beta diversity analysis included Principal Coordinates Analysis (PCoA) using Weighted UniFrac Distance Matrix Analysis and taxon proportions as input.

***Statistical analysis***

The analyses were conducted using the statistical software SigmaPlot 12.0 (Systat Software, San Jose, CA, USA), SPSS version 21 (IBM Brasil, SP), and JAMOVI version 2.4.7 (The Jamovi Project, Sydney, New South Wales, Australia). Periodontal, anthropometric, sociodemographic, and lifestyle parameters, as well as dietary frequency, bowel habits, and data from 3-day food records questionnaires were computed for each participant within the clinical groups and presented as frequency tables, mean, standard deviation, median, and interquartile range. Variables were assessed for normal distribution (Shapiro-Wilk test). Group comparisons were performed using ANOVA followed by Tukey's post hoc test when normality assumptions were met, or the Kruskal-Wallis test followed by Dunn's test when normality was not observed. The association between nominal qualitative variables was evaluated using the Chi-square test.

Regarding microbiological data, taxa assigned to species and/or phylotypes (with a frequency ≥10% and mean relative abundance of ≥0.1%) were used to calculate diversity metrics and correlation coefficients. Associations and differences between groups for these parameters were assessed using Chi-square, Mann-Whitney, Kruskal-Wallis, ANOVA, MANCOVA, and Spearman correlation tests. For alpha diversity, statistical significance among the 3 groups was evaluated using Kruskal-Wallis test, and Mann-Whitney was used for differences between group pairs. Permutational Multivariate Analysis of Variance (PERMANOVA) was used to quantify beta diversity differences between groups. Graphical presentation of data was generated using the QIIME2 Emperor tool to explore bacterial community structure. Stepwise Multiple Discriminant Analysis (MDA) using the Mahalanobis distance metric was conducted to predict whether a patient could be classified as having PE, GG or PH, based on a set of oral-gut species, socio-demographic, nutritional and anthropometric parameters as predictor variables. Predictor variables entered into the model included 48 oral and 22 gut taxa identified at the species/phylotypes, as well as age, income, anthropometric measurements and dietary data. Variables were log transformed and standardized as z-scores for calculations. A significance level of 5% was adopted for all analyses.

**RESULTS**

***Oral-fecal microbiota composition***

The relative abundance of phyla among groups in oral and fecal samples is depicted in **Figure S2**. Considering all clinical groups, the most abundant phyla in fecal samples were *Bacillota* (50%) and *Bacteroidota* (37.7%) (**Figure S2 B**), while in biofilm samples (**Figure S2 A**), the phyla *Fusobacteriota* (43%) and *Bacillota* (23%) predominated. Comparisons among the clinical groups demonstrated that the phyla *Bacteroidota* and *Pseudomonadota* were significantly reduced in fecal samples from patients with PE (**Figure S2 B**). In oral samples (**Figure S2 A**), the phyla *Pseudomonadota* and *Actinomycetota* were decreased in sites with periodontal pockets compared to sites with healthy periodontium, gingivitis, and/or healthy sites in PE (p<0.05, Kruskal-Wallis and Mann-Whitney tests). The phylum *Pseudomonadota* was significantly increased in oral and fecal samples of healthy sites and/or patients (p<0.01).

Regarding species/phylotypes in the fecal microbiota, significant differences between groups were seen for a few taxa, after controlling for age, income, education, anthropometric and nutritional parameters. *Fastidiosipila sanguinis* and *Ruminococcaceae [G-2] bacterium* HMT 085 were more enriched in fecal samples of PE patients than individuals with GG and PH, respectively (**Figure 3A**). In contrast, *Clostridiales [F-3][G-1] bacterium* HMT 876 was significantly reduced in GG compared to PH individuals (adjusted p<0.05, Bonferroni test, **Figure 3A**). In biofilm samples, species such as *Lautropia mirabilis*, *Corynebacterium matruchotii,* *Leptotrichia hongkongensis*, *Haemophilus parainfluenzae*, *Fusobacterium* sp. HMT203, and *Veillonella parvula* were significantly more abundant in healthy patients/sites than patients with PE (periodontal pockets) and/or GG, whereas *Fusobacterium nucleatum* subsp. *animalis, Porphyromonas endodontalis, Porphyromonas gingivalis,* and *Leptotrichia* sp. *HMT498* predominated in PE (periodontal pockets) samples, after controlling for other covariables (adjusted p<0.05; **Figure 3B**).

***Correlation between Oral and Fecal Taxa***

In PH, oral *Porphyromonas endodontalis* correlated with fecal *Streptococcus salivarius* and *Lachnospiraceae [G-2] HMT 088*; in GG, with *Bifidobacterium longum*; and in PE, with *Alloprevotella* sp. HMT 308. Oral *Capnocytophaga* sp. HMT 326 was linked to fecal *A. rava* (PH) and to *Lachnospiraceae HMT 088* and *Megasphaera* sp. HMT 841 (GG). Oral *Kingella oralis* correlated with fecal *Escherichia coli* in PH and GG. Oral *Leptotrichia* sp. HMT 225 was associated with fecal *Slackia exigua* in PH and PE. Other correlations included oral *Bacteroidales* HMT 274 with fecal *Slackia exigua* (GG) and *Bacteroides pyogenes* (PE), and oral *Prevotella* sp. HMT 313 with fecal *Alloprevotella* sp. *HMT 308* (PH) and *Clostridiales HMT 402* (PE). *Streptococcus oralis* subspecies showed consistent correlations with fecal *HMT 899* and *Streptococcus salivarius* in GG and PE (**Table S2**).

**REFERENCES**

Callaway, C. W., et al. (1988). Circumferences. In T. G. Lohman, A. F. Roche, & R. Martorell (Eds.), *Anthropometric standardization reference manual* (pp. 39-54). Champaign: Human Kinetics Books.

Chapple, I. L. C., Mealey, B. L., Van Dyke, T. E., Bartold, P. M., Dommisch, H., Eickholz, P., Geisinger, M. L., Genco, R. J., Glogauer, M., Goldstein, M., Griffin, T. J., Holmstrup, P., Johnson, G. K., Kapila, Y., Lang, N. P., Meyle, J., Murakami, S., Plemons, J., Romito, G. A., … Yoshie, H. (2018). Periodontal health and gingival diseases and conditions on an intact and a reduced periodontium: Consensus report of workgroup 1 of the 2017 World Workshop on the Classification of Periodontal and Peri‐Implant Diseases and Conditions. *Journal of Clinical Periodontology*, *45*(S20).<https://doi.org/10.1111/jcpe.12940>

Lourenço, T. G. B., de Oliveira, A. M., Tsute Chen, G., & Colombo, A. P. V. (2022). Oral‐gut bacterial profiles discriminate between periodontal health and diseases. *Journal of Periodontal Research*, *57*(6), 1227–1237. https://doi.org/10.1111/jre.13059

Nascimento, M. V. M. R. do, Silvestre, M. P. S., Costa, A. L. A. da, Lopes, M. B., Lourenço, T. G. B., & Posch, A. T. (2020). Nutritional influences on oral infections: the oral microbiota modulation. *Rio de Janeiro Dental Journal (Revista Científica Do CRO-RJ)*, *5*(2), 2–15. https://doi.org/10.29327/24816.5.2-2

Papapanou, P. N., Sanz, M., Buduneli, N., Dietrich, T., Feres, M., Fine, D. H., Flemmig, T. F., Garcia, R., Giannobile, W. V., Graziani, F., Greenwell, H., Herrera, D., Kao, R. T., Kebschull, M., Kinane, D. F., Kirkwood, K. L., Kocher, T., Kornman, K. S., Kumar, P. S., … Tonetti, M. S. (2018). Periodontitis: Consensus report of workgroup 2 of the 2017 World Workshop on the Classification of Periodontal and Peri‐Implant Diseases and Conditions. *Journal of Periodontology*, *89*(S1). https://doi.org/10.1002/JPER.17-0721

World Health Organization. (1995). *Physical status: The use and interpretation of anthropometry*. Geneva: WHO.

World Health Organization. (2011). *Waist circumference and waist-hip ratio: Report of a WHO expert consultation*. Geneva: World Health Organization. Retrieved from<https://iris.who.int/handle/10665/44583>. Accessed May 31, 2024.

Zanchim, M. C., Kirsten, V. R., & Marchi, A. C. B. D. (2018). Dietary intake markers of diabetic patients evaluated using a mobile application. *Ciência & Saúde Coletiva, 23*, 4199-4208.
