# Supplementary figures and images for: The Interplay Between Lifestyle and Oral/Faecal Microbial Profiles Among Periodontal Disease Patients: A Cross‐Sectional Study
Source: J Clin Periodontol. 2025 Sep 7;53(1):82–97. doi: 10.1111/jcpe.70029 (PMC12695455; doi:10.1111/jcpe.70029)

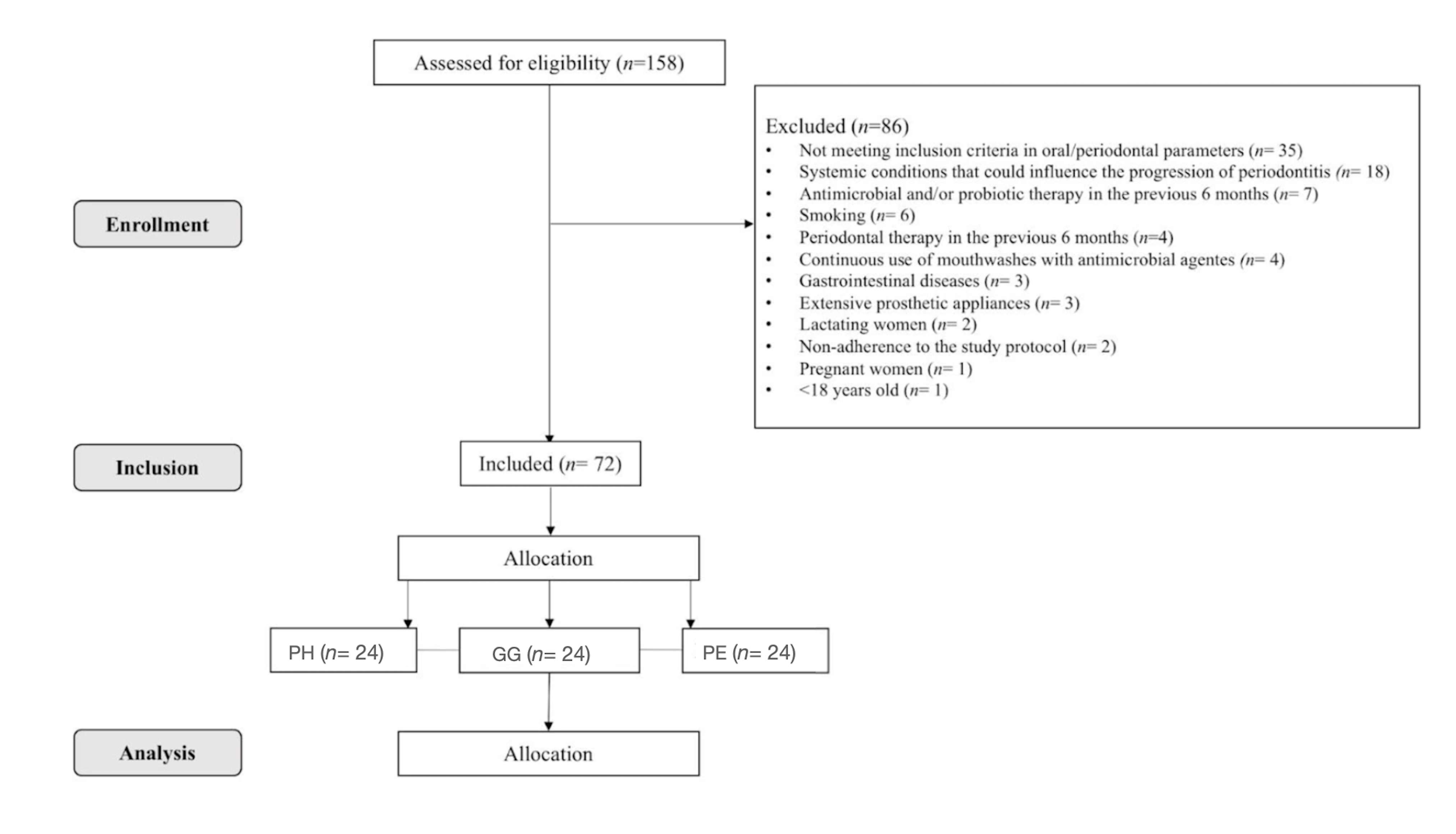

Supplement: Supplementary file 2 — Figure S1: Flowchart of the study design. [file JCPE-53-82-s004.tiff]

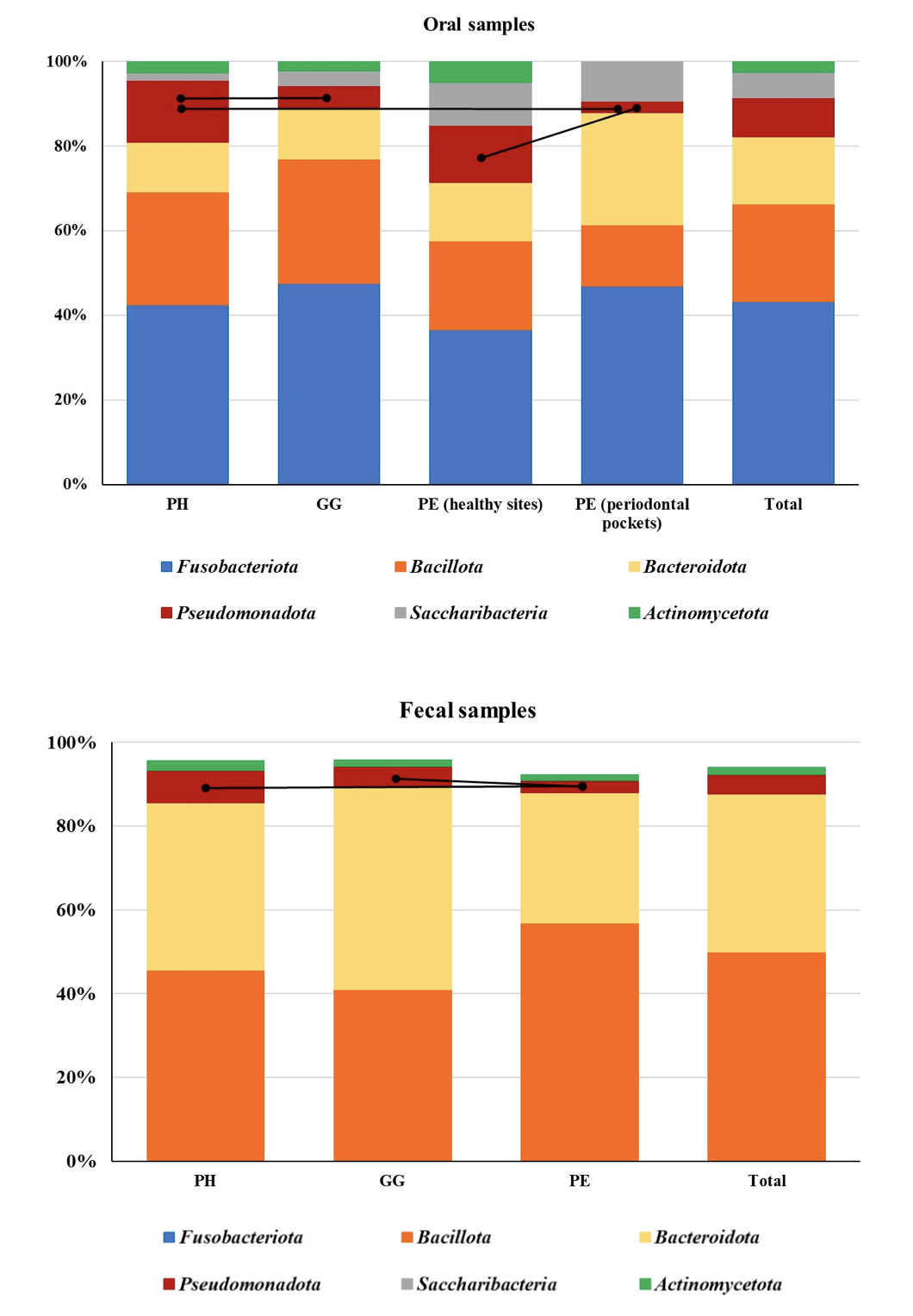

Supplement: Supplementary file 3 — Figure S2: Relative abundance of the most prevalent phyla (≥ 1.0% of the mean relative abundance across all samples) in oral (A) and faecal (B) samples within clinical groups. The bars indicate significant differences between groups for faecal and oral samples (Kruskal–Wallis and Mann–Whitney tests, p < 0.01). [file JCPE-53-82-s003.tiff]
