# Supplementary material for: The Interplay Between Lifestyle and Oral/Faecal Microbial Profiles Among Periodontal Disease Patients: A Cross‐Sectional Study
Source: J Clin Periodontol. 2025 Sep 7;53(1):82–97. doi: 10.1111/jcpe.70029 (PMC12695455; doi:10.1111/jcpe.70029)
Supplement: Supplementary file 4 — Table S1: Dietary habits data from the Food Frequency Questionnaire (FFQ) and evacuatory habits of individuals from groups PH, GG and PE. [file JCPE-53-82-s005.docx]

**Table S1.** Dietary habits data from the Food Frequency Questionnaire (FFQ) and evacuatory habits of individuals from groups Periodontal Health (PH), Gingivitis (GG) and Periodontitis (PE).

| **Parameters** | **PH**  **(*n* = 24)** | **GG**  **(*n* = 24)** | **PE**  **(*n* = 24)** |
| --- | --- | --- | --- |
| Raw salad {(median (IQR)} | 2.0 (1.0 - 3.0) | 2.0 (1.0 - 2.75) | 2.0 (1.0 - 3.0) |
| Cooked vegetables {(median (IQR)} | 2.0 (1.0 - 3.0) | 2.0 (1.0 - 3.0) | 2.0 (1.0 - 3.0) |
| Fresh fruits or fruit salad {(median (IQR)} | 3.0 (1.0 - 3.0) | 3.0 (1.0 - 3.0) | 2.0 (1.0 - 3.0) |
| Beans {(median (IQR)} | 2.0 (1.0 - 3.0) | 3.0 (2.0 - 3.0) | 3.0 (1.25 - 3.0) |
| Milk or yogurt {(median (IQR)} | 1.5 (1.0 - 3.0) | 2.5 (1.0 - 3.0) | 1.5 (1.0 - 3.0) |
| Potato chips, packaged, fried snacks{(median (IQR)} | 1.0 (1.0 - 1.0) | 1.0 (1.0 - 1.0) | 1.0 (1.0 - 1.0) |
| Salty crackers or packaged snacks {(median (IQR)} | 1.0 (1.0 - 1.0) | 1.0 (1.0 - 1.0) | 1.0 (1.0 - 1.0) |
| Hamburger and processed meats {(median (IQR)} | 1.0 (1.0 - 1.75) | 1.0 (1.0 - 2.0) | 1.0 (1.0 - 2.0) |
| Sweets in general {(median (IQR)} | 1.0 (1.0 - 3.0) | 2.0 (1.0 - 2.75) | 2.0 (1.0 - 3.0) |
| Soda{(median (IQR)} | 1.0 (1.0 - 1.0) | 1.0 (1.0 - 2.75) | 1.0 (1.0 - 3.0) |
| Red and/or white meat {(median (IQR)} | 3.0 (2.0 - 3.0) | 3.0 (3.0 - 3.0) | 3.0 (2.0 - 3.0) |
| Alcoholic beverage {(median (IQR)} | 1.0 (1.0 - 1.0) | 1.0 (1.0 - 1.0) | 1.0 (1.0 - 1.0) |
| Evacuation frequency {(median (IQR)} | 2.0 (1.25 - 3.0) | 2.0 (2.0 - 2.0) | 2.0 (1.25 - 2.0) |
| Pain during evacuation(%)  Yes  No | 4.2  95.9 | 4.2  95.9 | 8.3  91.7 |
| Straining when evacuating (%)  Yes  No | 12.5  87.5 | 33.3  66.7 | 16.7  83.3 |
| Bristol stool scale(%)  Type 1  Type 2  Type 3  Type 4  Type 5  Type 6  Type 7 | 0.0  12.5  50.0  25.0  8.3  4.2  0.0 | 4.2  4.2  45.8  37.5  4.2  0.0  4.4 | 8.3  4.2  45.8  33.3  4.2  4.2  0.0 |

The dietary data obtained from the FFQ were categorized as 1: rarely (0-1 day/week); 2: occasionally (2-4 days/week) and 3: frequently (≥5 days/week) (Ferreira et al.. 2010). Bowel movement frequency was categorized as once a day (1). more than once a day (2). and every two days or more (3). IQR: Interquartile range. Bristol Stool Scale (Lewis; Heaton. 1997)
