# Supplementary material for: The Interplay Between Lifestyle and Oral/Faecal Microbial Profiles Among Periodontal Disease Patients: A Cross‐Sectional Study
Source: J Clin Periodontol. 2025 Sep 7;53(1):82–97. doi: 10.1111/jcpe.70029 (PMC12695455; doi:10.1111/jcpe.70029)
Supplement: Supplementary file 5 — Table S2: Spearman's correlation (rho) analysis between oral and faecal bacterial species in each clinical group. (a) Significant correlations (ρ > 0.500; p < 0.01)—periodontal health (PH). (b) Significant correlations (𝜌 > 0.500; p < 0.01)—gingivitis (GG). (c) Significant correlations (ρ > 0.500; p < 0.01)—periodontitis (PE). [file JCPE-53-82-s002.docx]

**Table S2**. Spearman's correlation (rho) analysis between oral and fecal bacterial species in each clinical group.

## Table S2a. Significant correlations (rho > 0.500; p < 0.01) – Periodontal Health (PH).

|  | **FECAL TAXA** | | | | | | | | | | | | | | | | | | | | | |
| --- | --- | --- | --- | --- | --- | --- | --- | --- | --- | --- | --- | --- | --- | --- | --- | --- | --- | --- | --- | --- | --- | --- |
|  | **PH** | | | | | | | | | | | | | | | | | | | | | |
|  | *C.bacteriumHMT402* | *Alloprevotella* sp*. HMT308* | | *S. salivarius* | | *S. exigua* | | *E. coli* | | *E. catenaformis* | *A. rava* | *L. bacterium HMT088* | *D. fairfieldensis* | *B. longum* | *B. bacterium HMT899* | *B. pyogenes* | *M. oralis* | *B. heparinolyticus* | *R. bacterium HMT085* | *Megasphaera* sp*. HMT841* | *Butyrivibrio* sp*. HMT080* |  |
| **ORAL TAXA** | | | | | | | | | | | | | | | | | | | | | |  |
| *P. endodontalis* |  |  | | **.722** | |  | |  | |  |  | **.603** |  |  |  |  |  |  |  |  |  |  |
| *Capnocytophaga* sp. *HMT326* |  |  | |  | |  | |  | |  | **.722** |  |  |  |  |  |  |  |  |  |  |  |
| *K. oralis* |  |  | |  | |  | | **.543** | |  |  |  |  |  |  |  |  |  |  |  |  |  |
| *Leptotrichia* sp*. HMT225* |  |  | |  | | **.798** | |  | |  |  |  |  |  |  |  |  |  |  |  |  |  |
| *Prevotella* sp*. HMT313* |  | **.660** | |  | |  | |  | |  |  |  |  |  |  |  |  |  |  |  |  |  |
| *F. nucleatum_subs polymorphum* | **.538** |  | |  | |  | |  | |  |  |  |  |  |  |  |  |  |  |  |  |  |
| *L. hongkongensis* | **.550** |  | |  | |  | |  | |  |  |  |  |  |  |  |  |  |  |  |  |  |
| *Prevotella* sp*. HMT472* |  |  | |  | |  | |  | |  |  | **.650** |  |  |  |  |  |  |  |  |  |  |
| *P. melaninogenica* |  |  | |  | | **.722** | |  | |  |  |  |  |  |  |  | **.660** |  |  |  |  |  |
| *V. parvula* |  |  | |  | |  | |  | |  |  |  | **.700** |  |  |  |  |  |  |  |  |  |
| *V. dispar* |  |  | |  | | **.660** | |  | |  |  |  |  |  |  |  |  |  |  |  |  |  |
|  | | | | | | | | | | | | | | | | | | | | | |  |

## Table S2b. Significant correlations (rho > 0.500; p < 0.01) – Gingivitis (GG).

|  | **FECAL TAXA** | | | | | | | | | | | | | | | | | | | | | |
| --- | --- | --- | --- | --- | --- | --- | --- | --- | --- | --- | --- | --- | --- | --- | --- | --- | --- | --- | --- | --- | --- | --- |
|  | **GG** | | | | | | | | | | | | | | | | | | | | | |
|  | *C.bacteriumHMT402* | *Alloprevotella* sp*. HMT308* | | *S. salivarius* | | *S. exigua* | | *E. coli* | | *E. catenaformis* | *A. rava* | *L. bacterium HMT088* | *D. fairfieldensis* | *B. longum* | *B. bacterium HMT899* | *B. pyogenes* | *M. oralis* | *B. heparinolyticus* | *R. bacterium HMT085* | *Megasphaera* sp*. HMT841* | *Butyrivibrio* sp*. HMT080* |  |
| **ORAL TAXA** | | | | | | | | | | | | | | | | | | | | | |  |
| *P. endodontalis* |  |  | |  | |  | |  | |  |  |  |  | **.500** |  |  |  |  |  |  |  |  |
| *Capnocytophaga* sp*. HMT326* |  |  | |  | |  | |  | |  |  | **.723** |  |  |  |  |  |  |  | **.657** |  |  |
| *K. oralis* |  |  | |  | |  | | **.549** | |  |  | **.604** |  |  |  |  |  |  |  |  |  |  |
| *S. oralis* subsp. *dentisani clade058* |  |  | |  | |  | |  | |  |  |  |  |  | **.549** |  |  |  |  |  |  |  |
| *Bacteroidales [G-2] bacterium HMT274* |  |  | |  | | **.549** | |  | |  |  |  |  |  |  |  |  |  |  |  |  |  |
| *F. nucleatum subs vincentii* |  |  | |  | |  | |  | |  |  | **.535** |  |  |  |  |  |  |  |  |  |  |
| *F. nucleatum subs animalis* |  |  | | **.723** | |  | |  | |  |  |  |  |  |  |  |  |  |  |  |  |  |
| *Leptotrichia* sp*. HMT498* |  |  | |  | |  | |  | |  | **.549** |  |  |  |  |  |  |  |  |  |  |  |
| *L. buccalis* |  | **.604** | |  | |  | |  | |  |  |  |  |  |  |  |  |  |  | **.549** |  |  |
| *Leptotrichia* sp*. HMT392* |  |  | |  | | **.723** | |  | |  |  |  |  |  |  |  |  |  |  |  |  |  |
| *P. intermedia* |  |  | |  | |  | |  | | **.500** |  |  |  |  | **.797** |  |  |  |  |  |  |  |
| *Saccharibacteria (TM7) [G-5] bacterium HMT356* |  |  | |  | |  | |  | |  |  |  |  |  |  |  |  |  |  | **.604** |  |  |

|  | | | | | | | | | | | | | | | | | | | | | |
| --- | --- | --- | --- | --- | --- | --- | --- | --- | --- | --- | --- | --- | --- | --- | --- | --- | --- | --- | --- | --- | --- |

## Table S2c. Significant correlations (rho > 0.500; p < 0.01) – Periodontitis (PE).

|  | **FECAL TAXA** | | | | | | | | | | | | | | | | | | | | | |
| --- | --- | --- | --- | --- | --- | --- | --- | --- | --- | --- | --- | --- | --- | --- | --- | --- | --- | --- | --- | --- | --- | --- |
|  | **PE** | | | | | | | | | | | | | | | | | | | | | |
|  | *C.bacteriumHMT402* | *Alloprevotella* sp*. HMT308* | | *S. salivarius* | | *S. exigua* | | *E. coli* | | *E. catenaformis* | *A. rava* | *L. bacterium HMT088* | *D. fairfieldensis* | *B. longum* | *B. bacterium HMT899* | *B. pyogenes* | *M. oralis* | *B. heparinolyticus* | *R. bacterium HMT085* | *Megasphaera* sp*. HMT841* | *Butyrivibrio* sp*. HMT080* |  |
| **ORAL TAXA** | | | | | | | | | | | | | | | | | | | | | |  |
| *P. endodontalis* |  | | **.553** | |  | |  | |  |  |  |  |  |  |  |  |  |  |  |  |  |  |
| *Leptotrichia* sp*. HMT225* |  | |  | |  | | **.533** | |  |  |  |  |  |  | **.590** |  |  |  |  |  |  |  |
| *Prevotella* sp*. HMT313* | **.506** | |  | |  | |  | |  |  |  |  |  |  |  |  |  |  |  |  |  |  |
| *S. oralis* subsp. *tigurinus clade070* |  | |  | | **.706** | |  | |  |  |  |  |  |  |  |  |  |  |  |  |  |  |
| *Bacteroidales[G-2] bacterium HMT274* |  | |  | |  | |  | |  |  |  |  |  |  |  | **.512** |  |  |  |  |  |  |
| *Fusobacterium* sp*. HMT203* |  | |  | |  | |  | |  |  |  |  |  | **.699** |  |  |  |  |  |  |  |  |
| *P. oulorum* |  | |  | |  | |  | |  |  |  |  |  | **.699** |  |  |  |  |  |  |  |  |
| *Saccharibacteria (TM7)[G-1] bacterium HMT348* |  | |  | |  | |  | |  |  |  |  | **.653** |  |  |  |  |  |  |  |  |  |
| *S. sanguinis* |  | |  | |  | |  | |  |  |  |  |  |  | **.699** |  |  |  |  |  |  |  |
|  |  | |  | |  | |  | |  |  |  |  |  |  |  |  |  |  |  |  |  |  |
|  |  | |  | |  | |  | |  |  |  |  |  |  |  |  |  |  |  |  |  |  |
|  |  | |  | |  | |  | |  |  |  |  |  |  |  |  |  |  |  |  |  |  |
